# 

**Published:** 1806-11

**Authors:** 


### NEW MEDICAL PUBLICATIONS.

Observations on the Nature, Kinds, Causes, and Prevention of Insanity. By Thomas Arnold. 8vo. 6s. boards.

Malvern Waters, being a re-publication of Cases formerly collected by John Wall, M. D. and since illustrated with Notes, &c. by his Son, Martin Wall, M. D.

Practical Observations on the principal Diseases of the Eyes, illustrated with Cases, translated from the Italian of Antonio Scarpa, Professor of Anatomy and Practical Surgery in the University of Pavia. By James Briggs, 10s. 6d. boards.

---

### *TO CORRESPONDENTS.*

Communications are received from Dr. Cayley, Dr. Thornton, Mr. Marson, Mr. Okes, Mr. Weldon, and Medicus.

